# The ageing immune system and its battle with viruses

**DOI:** 10.1099/jgv.0.002320

**Published:** 2026-08-24

**Authors:** Jessica A. Gaevert, Carolien E. van de Sandt

**Affiliations:** 1 Murdoch Children’s Research Institute, Parkville, Victoria, Australia; 2 St Jude Children’s Research Hospital Department of Host-Microbe Interactions, Memphis, Tennessee, USA; 3 Department of Microbiology and Immunology, University of Melbourne, at the Peter Doherty Institute for Infection and Immunity, Melbourne, Victoria, Australia

**Keywords:** immunosenescence, CD8^+ ^T cells, acute and chronic/latent viruses, ageing, vaccination, correlates of protection

## Abstract

The global increase in life expectancy has resulted in a growing proportion of older individuals at increased risk of severe disease outcomes following viral infections. CD8^+^ T cells play a vital role in controlling viral infections and provide protection against severe disease by eliminating virus-infected cells. Furthermore, CD8^+^ T cells recognize conserved internal viral proteins, enabling cross-reactivity and the formation of long-term immunological memory. However, a lifetime of exposures to acute and persistent latent viruses, vaccinations and the age-associated decline in immune functions (immunosenescence and inflammaging) have a profound impact on virus-specific CD8^+^ T cell populations. The age-associated progressive decline of naïve CD8^+^ T cells, reduced T cell receptor (TCR) diversity, the accumulation of highly differentiated memory and/or exhausted CD8^+^ T cells and low-level chronic inflammation directly affect virus-specific immunity. These age-associated changes impair both primary and recall CD8^+^ T cell responses to infections and vaccinations in older individuals. Both ageing and exposure history impact the CD8^+^ T cell correlates of protection, including frequency, phenotype, TCR diversity, polyfunctionality, cytotoxic potential and proliferation capacity. The implications of these changes are discussed in the context of acute (influenza and respiratory syncytial virus), persistent latent (cytomegalovirus, Epstein–Barr virus and varicella-zoster virus) and novel (severe acute respiratory syndrome coronavirus 2) viruses. An in-depth understanding of how lifelong viral exposures intersect with immunosenescence will elucidate how virus-specific CD8^+^ T cells change with age. These insights will aid vaccine strategies to effectively harness CD8^+^ T cells and induce long-lasting, broadly reactive virus-specific immunity.

## Graphical Abstract

Virus-specific immunity and correlates of protection change across human life: children encounter first infections/vaccinations when they are immunologically naïve, whereas cumulative exposure histories in older adults shape their immunity.

## Introduction

Effective immunity to viral infections is of critical importance in preventing severe morbidity and mortality. Specifically, our understanding of how the immune system changes in the context of ageing has become increasingly important. By 2050, ~22% of the world’s population will be over the age of 60, which is nearly double what it was in 2015 [1]. Furthermore, global demographic trends show that the pace of population ageing is accelerating [1]. The age-associated decline in immune functions, also known as immunosenescence, heightens the vulnerability of older adults to severe virus infections and reduces their response to vaccinations. This has profound implications for public health, vaccine design and pandemic preparedness [2]. Indeed, older adults experience increased morbidity and mortality from viral respiratory infections, including influenza viruses, respiratory syncytial virus (RSV) and severe acute respiratory syndrome coronavirus 2 (SARS-CoV-2) [3–6]. Furthermore, chronic and latent viral infections contribute to long-term remodelling of the immune system and thereby further accelerate the age-associated immune decline [7, 8].

The exact mechanisms underpinning immunosenescence are not fully understood [7, 9, 10]. Hallmarks of ageing immunity include (1) a loss of naïve T cells as a result of thymic involution [11–13], (2) an accumulation of memory T cells, including exhausted and senescent T cells, which display reduced functionality and expansion capacity during viral infections [14] and (3) reduced production of high-affinity antibodies by B cells [15, 16]. In addition, older individuals often experience chronic, low-grade systemic inflammation, also known as inflammaging, which makes many immune cell subsets less sensitive to acute challenges as we age [17, 18]. These impaired immune responses result in increased susceptibility to infections, autoimmunity and cancer [11–13].

CD8^+^ T cells eliminate virus-infected cells and generate long-term immunological memory. This means that CD8^+^ T cells play a key role in controlling viral infections and protection against severe disease outcomes [19–23]. Across a human lifetime, exposures to acute and chronic/persistent latent viruses shape the antiviral potential of the CD8^+^ T cell population [12, 24–26]. A deeper understanding of how virus-specific CD8^+^ T cell immunity changes across the human lifespan is essential for developing strategies to enhance antiviral immunity in ageing populations.

In this review, we provide an overview of our current knowledge on how virus-specific CD8^+^ T cell immunity evolves across the human lifespan. We explore the effect of ageing on CD8^+^ T cell-mediated protection against repeated acute, novel and persistent latent viral infections. Finally, we discuss if and how CD8^+^ T cells can be harnessed through vaccination to induce robust long-lasting virus-specific immunity in older individuals.

### Virus exposures across the human lifespan

**Fig. 1.**
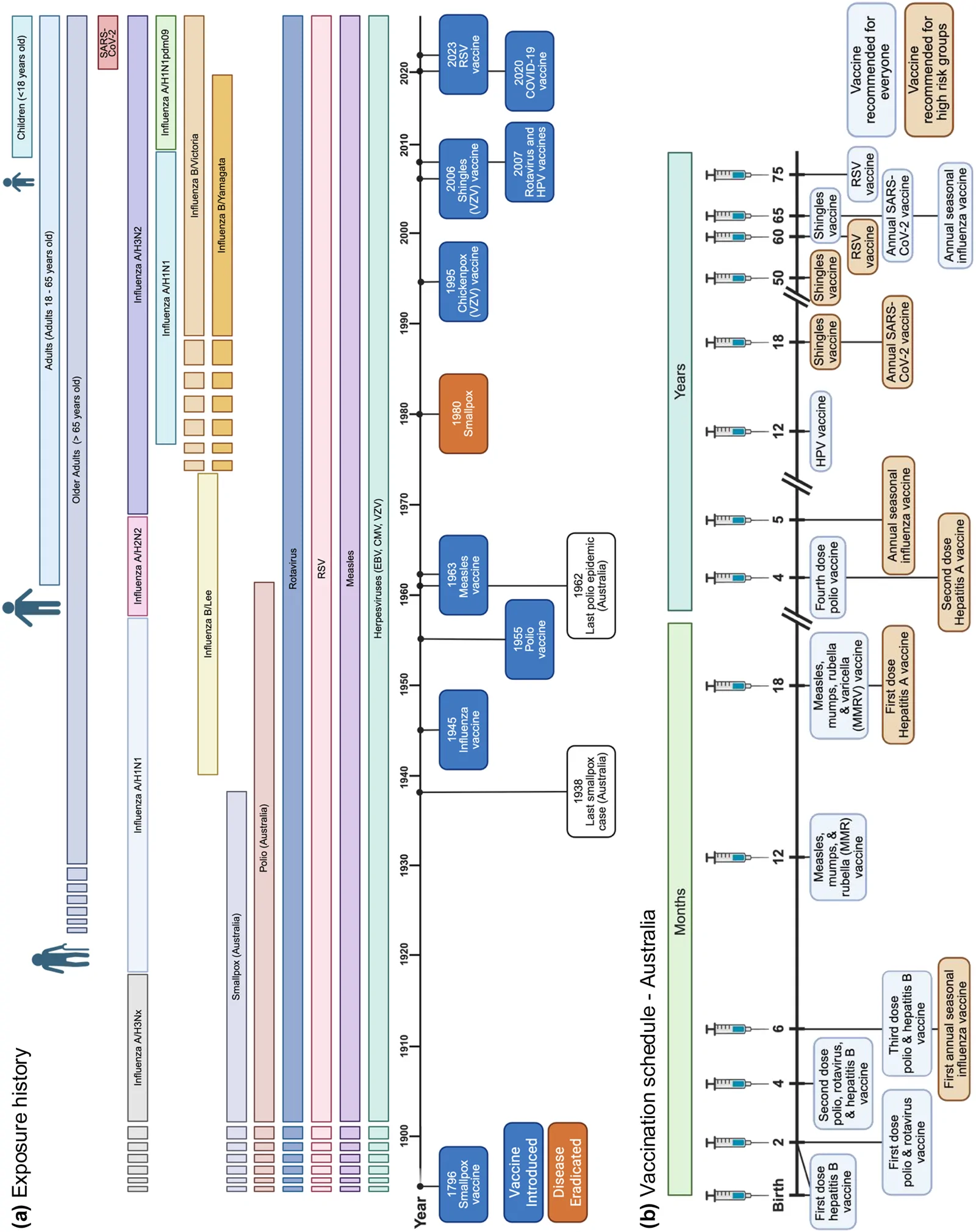
Timeline of infections and virus-specific vaccinations over a human lifetime (**a**) Timeline of the last century showing circulation of common viruses to which different age groups could have been exposed. Boxes below indicate the year of introduction of commonly used vaccines that the populations may have received, as well as last cases of smallpox and polio in Australia. (**b**) Australian immunization schedule for virus vaccines. Additional bacterial vaccines are also provided as part of the Australian immunization schedule but are not included in the figure. The full Australian immunization schedule can be found here [37]. Created in BioRender (https://BioRender.com).

The exposure to viral infections over the course of a human life shapes immune memory. The type of viral exposures and available vaccines differs between children, adults and older adults, who grew up in different time periods (Fig. 1a). Understanding which viral exposures occur in each period of life provides important context for analysing and interpreting age-specific immune responses.

First-time exposures to most viruses occur in the first decade of human life (0–10 years old). By 2 years of age, almost all children have been infected with RSV, and most individuals experience reinfections throughout their life [27–29]. First exposure to influenza A and B viruses typically occurs before the age of 6 [30, 31]. Early-life imprinting or antigenic sin refers to a phenomenon where the first influenza strain you encounter during childhood, either through infection or vaccination, affects lifelong influenza-specific antibody profiles [32–34]. Original antigenic sin may also occur in the context of other viral infections. One of the best-known other examples is following dengue virus infection, where prior exposure to one serotype can bias immune responses during subsequent infection with a heterologous serotype. In this setting, the recall of suboptimal antibody responses may contribute to antibody-dependent enhancement and increase the risk for severe disease [35]. Other frequently observed viral infections during early childhood are caused by parainfluenza, adenoviruses, rhinoviruses and rotavirus [36]. Childhood immunization programmes aim to prevent severe virus and bacterial infections early in life and establish immunological memory prior to first exposure [37]. The Australian national childhood immunization programme includes multiple vaccines to prevent virus-induced diseases, including the mumps, measles, rubella, rotavirus, varicella [varicella-zoster virus (VZV), chickenpox], polio and hepatitis B vaccines (Fig. 1b) [37]. Additional influenza and hepatitis A vaccinations are offered to high-risk groups early in life [37]. These early exposures and vaccinations collectively shape the foundation of the developing virus-specific memory in the B and T cell compartments.

In adolescence (10–19 years old) and adulthood (20–64 years old), individuals continue to be exposed to circulating respiratory viruses like RSV and influenza. In addition, first exposure to herpesviruses often occurs during this time of life and can result in lifelong latent infections. Seroprevalence for Epstein–Barr virus (EBV) increases in adolescence/early adulthood and by middle age, greater than 90% of the population show to be seropositive for EBV [38]. Cytomegalovirus (CMV) exposure is possible at any age, but seroprevalence increases with age and can be as high as 90% [39]. Yet, the level of seroprevalence for these and other herpes viruses also depends on geographic regions [40, 41]. Importantly, persistent latent CMV infections have been shown to directly affect CD4^+^ and CD8^+^ T cell differentiation, expansion and repertoire diversity [41, 42]. Adolescence is also a typical window for the human papillomavirus (HPV) vaccination, further adding to vaccine-induced immune memory [43].

As individuals age, they accumulate repeated exposures to the annually circulating viral infections listed above, including RSV and influenza A and B viruses, and experience increasing viral dynamics associated with latency and reactivation of herpesvirus infections [26, 29, 40–42]. Older adults have experienced numerous influenza exposures through infections and annual vaccinations across decades [44, 45], yet their risk for severe infections increases after the age of 65 [46, 47]. Annual influenza vaccination is therefore advised for individuals over the age of 65 (Fig. 1b) [37]. Reinfections with RSV are also common in adulthood and pose a particular problem in older adults [29]. Therefore RSV vaccination is now recommended for individuals over the age of 75 (Fig. 1b) [37]. Older adults may also experience painful and serious reactivation of VZV, known as shingles, which can be prevented by administering the recombinant VZV vaccine (Shingrix) (Fig. 1b) [48, 49]. The emergence of SARS-CoV-2 in 2020 caused a global pandemic, resulting in a first-time exposure to a novel virus in different age groups. Yet, the more severe disease outcomes were associated with increasing age [50]. Annual SARS-CoV-2 vaccination is highly recommended for older adults (>65 years) because these respiratory viral infections continue to pose serious risks and complications in this population (Fig. 1b) [37, 51]. Another point of note is that some other vaccines have been shown to generate extremely long-lived and durable immunity. The yellow fever vaccine, which is given to people living in or travelling to endemic regions, has been shown to induce immunity for longer than 30 years [52, 53]. Vaccines like this provide important insights into how we can successfully elicit and maintain long-lasting immune responses.

How the immune system responds to viral exposures differs distinctly over a lifetime. Children are mostly immunologically naïve when they experience first viral infections and vaccinations. Conversely, older adults have a cumulative exposure history composed of both acute and persistent latent infections and vaccinations, which have shaped their immune response. Together, these factors create fundamentally different antiviral immune landscapes, including CD8^+^ T cell responses, across the human lifespan.

### Diverse virus-specific CD8^+^ T cell subsets

While CD8^+^ T cells play a critical role in controlling viral infections, the CD8^+^ T population itself is highly heterogeneous and comprises multiple functionally distinct subsets. These CD8^+^ T cell subsets differ in their phenotypic markers, differentiation state and capacity to mediate virus-specific cytotoxic immune responses.

**Fig. 2.**
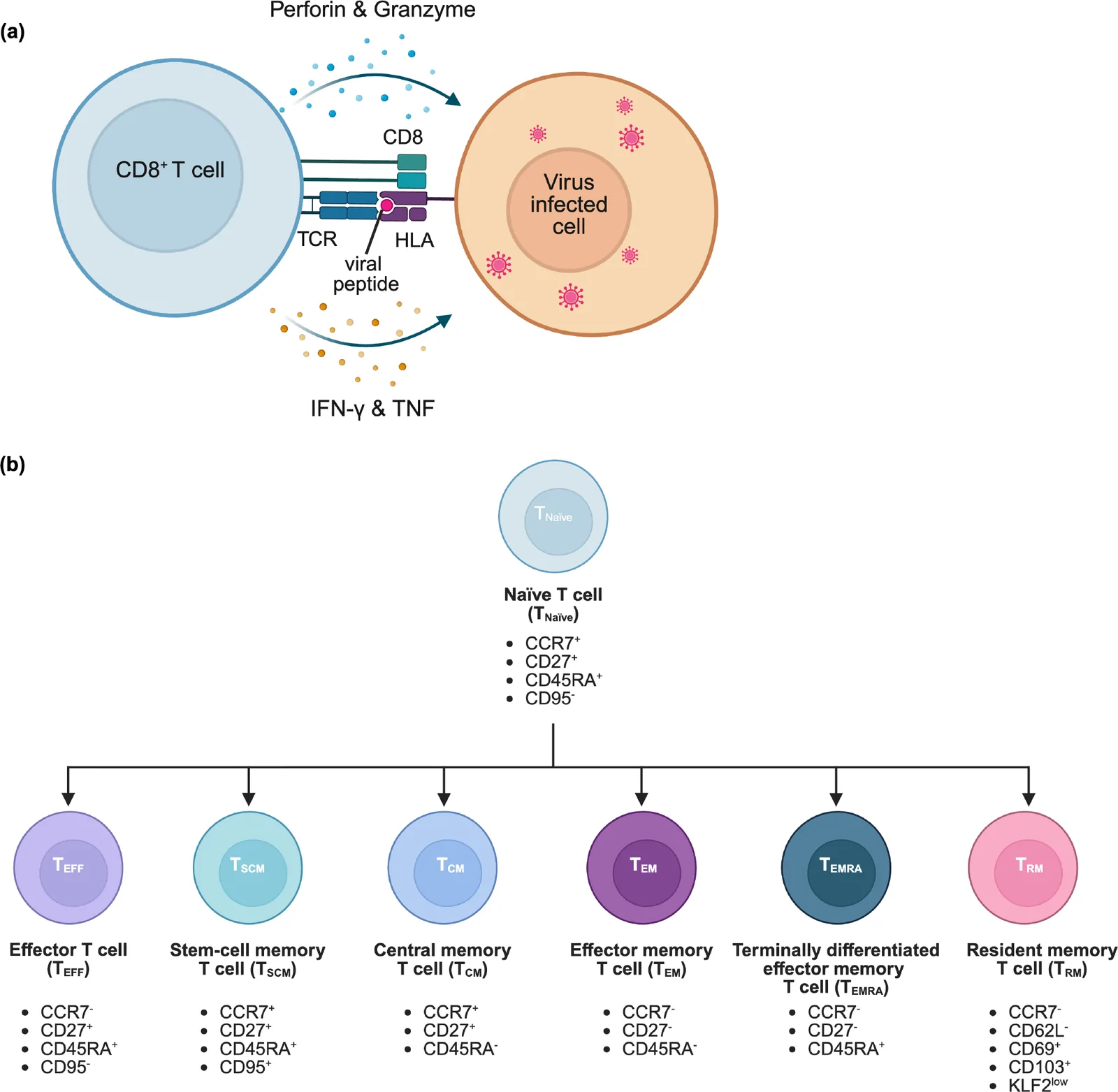
CD8^+^ T cell activation and differentiation into memory subsets. (**a**) CD8^+^ T cells use their TCR to recognize viral peptides presented by HLA class I molecules on the surface of a virus-infected cell. CD8 acts as a co-receptor for this process and can strengthen binding avidity. Specific recognition of a pHLA complex by the TCR results in the release of proinflammatory cytokines IFN-γ and TNF by the CD8^+^ T cell. In addition, the CD8^+^ T cell releases perforin and granzyme, which will kill the virus-infected cell. (**b**) Upon activation, naïve CD8^+^ T cells differentiate into effector and memory CD8^+^ T cell subsets. Created in BioRender (https://BioRender.com).

At birth, the CD8^+^ T cell population consists of naïve T cells (T_naïve_), which circulate in the blood, lymphoid organs and peripheral tissues. Naïve T cells typically display the canonical phenotype CCR7^+^, CD27^+^, CD45RA^+^ and CD95^−^ [54–57]. CD8^+^ T cells use their T cell receptor (TCR) to survey the environment for potential threats. TCRs recognize epitopes, small viral fragments called peptides (p) bound to a HLA class I molecule (pHLA-I) on the surface of infected or antigen-presenting cells (Fig. 2a). The high diversity of the human TCR repertoire, with ~10^12^ distinct TCRs within an individual, allows recognition of a broad range of viral epitopes (or antigens) [58–60]. Upon antigen-specific recognition, CD8^+^ T_naïve_ cells differentiate into distinct memory and effector subsets [57]. Effector T cells (T_EFF_; CCR7^−^, CD27^+^, CD45RA^+^ and CD95^−^) are characterized by downregulation of CCR7 and acquisition of cytotoxic functions [54–57]. Long-lived memory subsets include stem-cell memory T cells (T_SCM_; CCR7^+^, CD27^+^, CD45RA^+^ and CD95^+^), central memory T cells (T_CM_; CCR7^+^, CD27^+^ and CD45RA^−^), effector memory T cells (T_EM_; CCR7^−^, CD27^−^ and CD45RA^-^), tissue-resident memory T cells (T_RM_; CCR7^−^, CD62L^−^, CD69^+^, CD103^+^ and KLF2^low^) and terminally differentiated effector memory T cells (T_EMRA_; CCR7^−^, CD27^−^ and CD45RA^+^) (Fig. 2b) [54, 57, 61–63]. Several models have been proposed to describe the lineage relationship between CD8^+^ T cell effector and memory subsets [54, 61, 64] and are likely influenced by a combination of the type of antigen, antigen dose, strength of TCR binding, time of antigen exposure, inflammatory cues in the environment (including cytokines) and transcription factors including T-bet and Eomes. Together these factors shape the durability and quality of the resulting response [54, 61, 62]. T_RM_ cells reside primarily in tissues, such as the lung, skin and gut*,* without recirculation through blood [57, 63]. However, T_RM_ populations may undergo turnover and/or replacement over time [65]. T_RM_ cells in the lung are particularly important for early detection and elimination of recurring respiratory viruses, where rapid local responses can limit infections before systemic responses become engaged [66]. T_RM_ cells are often defined as CCR7^-^, CD62L^-^, CD69^+^, CD103^+^ and KLF2^low^. However, it is important to note that the expression of T_RM_ markers depends on the organ, stimulation history and environmental factors [57, 67, 68]. In contrast, T_SCM_ and T_CM_ circulate in blood and lymphoid organs, whereas T_EM_ and T_EMRA_ cells mainly migrate between blood and tissue environments [57, 61]. T_SCM_ are multipotent, and capable of self-renewal and differentiation into other memory subsets [69]. T_EMRA_ cells often develop after prolonged antigen exposure. T_EMRA_ cells may still exert rapid cytotoxic functions, yet their proliferation capacity is limited compared to other memory subsets [54–56]. While often discussed together, terminal differentiation, senescence and exhaustion represent distinct CD8^+^ T cell states [54, 56, 57]. Terminally differentiated (T_EMRA_) CD8^+^ T cells retain cytotoxic function but exhibit limited proliferative capacity [54, 56, 57]. Senescent T cells display impaired proliferative potential and are frequently associated with expression of markers CD57 and KLRG1 but can still carry out effector functions [56, 57]. Exhausted T cells arise primarily as a result of chronic antigen stimulation, resulting in progressive functional impairment in addition to the expression of inhibitory receptors including PD-1 [56, 57]. Although these states can share phenotypic and functional characteristics, they represent distinct biological processes and should not be considered interchangeable [56, 57].

The diversity and maintenance of these memory populations is critical to providing long-lasting and rapid immune responses when re-encountering the same virus, particularly in the context of antigenic drift and keeping chronic and/or persistent latent infections under control [70–72].

### Correlates of protection of virus-specific CD8^+^ T cells

Correlates of protection (COP) are measurable immune responses that predict protection from symptomatic infection or severe disease outcomes and are central to evaluating and defining virus-specific immunity. Understanding the COP for CD8^+^ T cells is critical for identifying individuals at high risk for severe disease outcomes and guiding vaccine and antiviral strategy development [73–75].

**Fig. 3.**
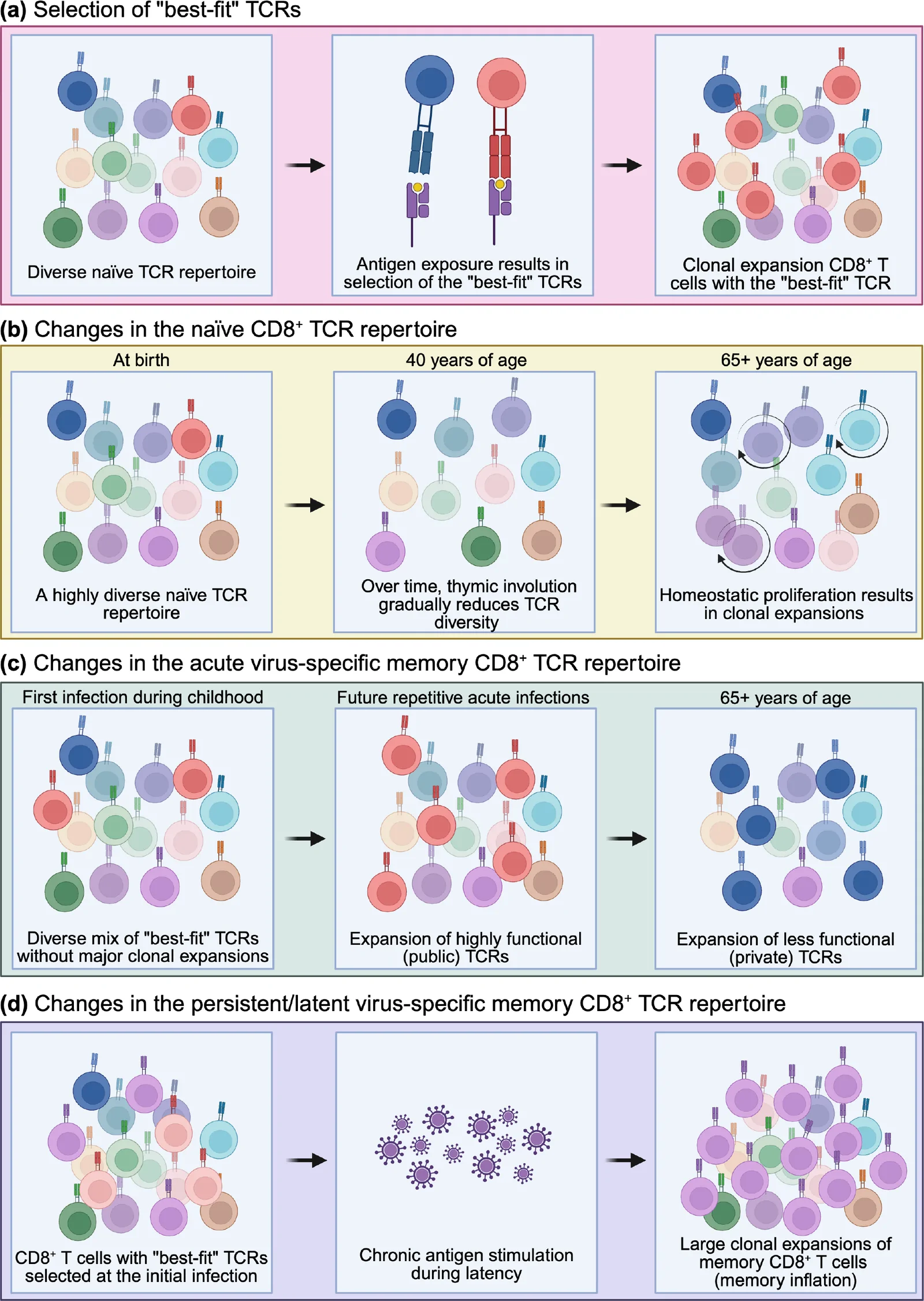
Age-related changes in the virus-specific TCR repertoire (**a**) CD8^+^ T cell with the ‘best-fit’ TCR are selected during first viral exposure based on their high avidity with the pHLA complex upon which they start to expand and differentiate. Their prevalence will further increase upon subsequent exposures with the same virus. (**b**) Changes in the naïve CD8^+^ TCR repertoire. The naïve CD8^+^ TCR repertoire is highly diverse at birth. Diversity gradually decreases due to thymic involution and cells being selected to go into the memory compartment. Once thymic function ceases, maintenance of the naïve CD8^+^ T cell population becomes dependent on homeostatic proliferation, resulting in clonal expansions and further narrowing of the naïve CD8^+^ TCR repertoire. (**c**) Changes in the TCR repertoire of acute virus-specific memory CD8^+^ T cells. Upon first infection during childhood, the memory CD8^+^ TCR repertoire consists of a diverse mix of ‘best-fit’ TCRs often without major clonal expansion and may include cells that express highly functional public TCRs. Studies of influenza A2/M1_58_-specific CD8^+^ T cells suggest that repeated exposures during childhood and adulthood drive further expansion of CD8^+^ T cells with highly functional (public) TCRs. Additionally, these ‘best-fit’ TCR expressing influenza A2/M1_58_-specific CD8^+^ T cells gradually disappear later in life and are replaced by clonally expanded CD8^+^ T cells expressing less-functional private TCRs. (**d**) Changes in the TCR repertoire of persistent latent virus-specific memory CD8^+^ T cells. While ‘best-fit’ TCR expressing CD8^+^ T cells are selected during the initial infection, chronic antigen stimulation during the latency phase drives memory inflation, resulting in large clonal expansions of memory CD8^+^ T cells expressing a particular TCR. Created in BioRender (https://BioRender.com).

During the first decade of life, naïve CD8^+^ T cells develop in the thymus where they undergo positive and negative selection, after which they replenish the circulating naïve CD8^+^ T cell population and preserve a highly diverse TCR repertoire [76, 77]. Naïve CD8^+^ T cells form the basis from which protective CD8^+^ T cell responses are generated against novel viral encounters or immune escape variants. A correlate of protection for primary immune responses is the size of the naïve population and its TCR diversity as together they determine the chance of recruiting high-quality CD8^+^ T cells [14]. After the first exposure to a virus, naïve CD8^+^ T cells expressing the ‘best-fit’ TCR, a TCR with a high avidity to the pHLA complex, are selected from the naïve TCR repertoire (Fig. 3a). Thus, a more diverse TCR repertoire within the naïve virus-specific CD8^+^ T cell population allows the generation of superior antiviral responses because it increases the potential for optimal TCR binding [24, 25, 78]. Memory CD8^+^ T cells expressing the ‘best-fit’ TCR often become more prevalent following subsequent encounters [25, 78–80]. Public TCRs are antigen-specific TCRs that are shared between unrelated individuals and are often enriched among effector and memory CD8^+^ T cell populations. Public TCRs are associated with optimal immunity in adults, due to their high TCR-pHLA affinity, ability to recognize peptide variants and higher T cell functionality [24, 25, 81]. In contrast, CD8^+^ T cells expressing private TCRs, which are unique to a single individual, tend to show reduced functional capacity and are less capable of recognizing peptide variants [25, 81, 82]. The naive CD8^+^ T cells detected in umbilical cord blood, indeed, express a highly diverse TCR repertoire [25]. However, the influenza-specific TCR repertoire at birth does harbour a bias towards public TCR features, which are commonly observed in memory populations later in life [25]. This bias was not observed for EBV or CMV-specific naïve CD8^+^ T cells in the cord blood [83]. Thomas and Crawford suggested that there may be an evolutionary advantage to having a naïve TCR repertoire that is biased towards recognizing prominent, common antigens that you encounter early in life (such as circulating influenza viruses) [84].

Virus-specific CD8^+^ T cell precursor frequency is another COP during first viral encounters. Higher naïve precursor frequencies are associated with immunodominant CD8^+^ T cell responses following primary infection [85]. Furthermore, higher frequency of virus-specific CD8^+^ T cells prior to exposure are associated with faster viral clearance and milder disease outcomes [19, 86]. Yet a threshold of precursor frequencies, or any virus-specific CD8^+^ T cell subset, associated with reduced disease severity remains ill defined. Thus far, only a threshold of >100 spot-forming units per million PBMCs has been proposed as the minimum frequency required for effective cell-mediated protection against symptomatic influenza infection in children [87].

Functional features of the responding CD8^+^ T cells, particularly polyfunctionality, cytotoxicity and proliferative capacity, provide additional COP to the virus-specific CD8^+^ T cell population [88]. As CD8^+^ T cells transition into the memory phase, the extent of these qualitative features often becomes subset-specific.

T_SCM_ and T_CM_ cells contribute to the proliferative reserve and breadth of recall responses, including their ability to rapidly differentiate into new effector cells upon re-exposure [14] and are associated with viral clearance [72, 89].

CD8^+^ T cell polyfunctionality is characterized by the cell’s ability to simultaneously produce antiviral cytokines such as IFN-γ, TNF and IL-2. These cytokines enhance viral control by promoting effector differentiation, expansion and survival of responding CD8^+^ T cells and by creating an antiviral environment in infected tissues, resulting in the recruitment and activation of other immune cells [76, 90]. Furthermore, cytotoxic functions, mediated primarily through granzyme B and perforin, or FAS ligand are essential for eliminating infected cells. Studies have shown that the expression of granzyme B and perforin is strongly associated with reduced disease severity [73, 74]. The high polyfunctionality of T_EM_ cells allows them to exert rapid effector function within hours of encountering an antigen [62], resulting in rapid control of acute infections [91, 92]. Because CD8^+^ T_EM_ populations primarily reside in the circulation, protection at the site of infection relies heavily on CD8^+^ T_RM_ responses, especially for respiratory viruses [62, 66]. T_RM_ cells reside in tissues and are essential for rapid recognition and elimination of virus-infected cells at the site of infection. Additionally, cytokines secreted by T_RM_ further escalate the immune response by attracting other immune cells from the circulation and make the local environment less permissive for pathogen replication [93]. However, a recent study by Lam *et al*. used retrospective radiocarbon (^14^C) birth dating to demonstrate that T_RM_ populations exhibit continuous turnover, with a mean lifespan of 1–2 years [65]. These findings suggest that T_RM_ populations may require frequent boosting to be maintained long-term.

### Age-related changes in virus-specific CD8^+^ T cells directly affect COP

As we age, the CD8^+^ T cell population undergoes age-related changes that greatly affect their ability to generate or reactivate virus-specific immunity later in life [7, 26, 94].

Over a lifetime, the thymus gradually shrinks and undergoes structural and functional decline, also known as thymic involution. This age-related gradual loss of thymic function reduces the output of naïve CD8^+^ T cells throughout adulthood, until it ceases almost entirely by the age of 40, although low levels of thymic output may persist till later in life [7, 26]. This results in a sharp decline of both circulating naïve CD8^+^ T cells and TCR diversity (Fig. 3b) [24, 94]. As thymic export diminishes, the naïve CD8^+^ T cell compartment becomes increasingly dependent on homeostatic proliferation to maintain numbers [95–98]. This process promotes clonal expansion and progressively declines the diversity within the TCR repertoire (Fig. 3b) [24, 96, 99]. This loss of TCR diversity may reduce the likelihood of recruiting high-quality CD8^+^ T cells during a first viral encounter later in life and may result in diminished capacity of older adults to mount robust *de novo* CD8^+^ T cell responses to emerging pandemic viruses (Fig. 3b). Indeed, Messaoudi *et al*. used a mouse model to demonstrate that lower TCR diversity increased susceptibility to viral infection, and their findings suggested that a broader TCR repertoire is important for recruiting high-avidity (best-fit) T cells capable of containing viral replication [100]. Furthermore, we and others have reported a gradual disappearance of ‘best-fit’, high-avidity public TCRs among virus-specific memory CD8^+^ T cells in older individuals [22, 24, 25]. While age-associated loss of TCR diversity and repertoire remodelling may reduce the likelihood of recruiting highly functional CD8^+^ T cell clonotypes during viral infection, a direct link between these age-related changes in the TCR repertoire and clinical disease severity has yet to be established in humans.

Ageing also leads to reduced polyfunctionality and reduced proliferative capacity, which slows down recall kinetics [26]. This contributes to delayed viral clearance upon reinfection [100–102]. CD8^+^ T_EM_ cells in older adults show diminished ability to co-produce IFN-γ, TNF and IL-2 [26]. Further, ageing increases the proportion of terminally differentiated and senescence-associated CD8^+^ T cell populations, including increase of phenotypic T_EMRA_ cells and T_EM_ cells expressing CD57 and/or KLRG1 [10, 14, 26]. These cells show preserved cytotoxicity but impaired expansion capacity [10, 14] and a reduced ability to sustain long-term memory [14, 26].

With age, the frequency of circulating virus-specific CD8^+^ T_CM_ populations may either decline, increase or remain stable depending on the type of virus [8, 14, 25, 64]. T_CM_ populations in older adults also exhibit transcriptional shifts, which lead to reduced self-renewal and increased stress-response signatures [64]. Functionally, T_CM_ in older individuals are less responsive to secondary infection or boosting via vaccination which has implications for vaccine designs [22, 23, 25]. In older adults, T_RM_ are also subject to detrimental changes due to ageing [94]. Specifically, ageing in T_RM_ is linked to dysregulated cytokine production and impaired reactivation [103, 104]. Disruption of T_RM_ homeostasis in the lungs due to ageing compromises local protection and contributes to immunopathology during RSV infection [103, 104].

Together, these age-specific changes within the CD8^+^ T cell population will need to be taken into account when developing standardized assays to measure COP and when designing novel vaccine strategies that aim to boost the CD8^+^ T cell population. While ageing has a clear impact on the COP, it is essential that we recognize that these COP are not fixed and in addition also vary between viruses. These virus-specific differences reflect the diverse ways our immune system engages with each virus.

### CD8^+^ T cell immunity to repetitive acute viral infections

Repetitive acute infections are often the result of inadequate humoral immunity. In the absence of effective neutralizing antibodies, CD8^+^ T cells play a central role in controlling repetitive acute viral infections and are associated with faster viral clearance and reduced disease severity [19, 21, 91, 92, 105].

Influenza provides one of the best-studied examples of CD8^+^ T cell-mediated control of repetitive acute viral infections. Seasonal influenza viruses gradually escape from the existing antibody response through the accumulation of mutations in their surface proteins (Heamagglutinin (HA) and Neuraminidase (NA)), a process also known as antigenic drift [106–108]. As a result of gradual antigenic drift, adults experience about two influenza virus infections every decade, while infections occur more frequently in children and adolescents [45]. Occasionally, antigenically distinct influenza A subtypes can be introduced into the human population in a process called antigenic shift (i.e. through zoonotic transmission and/or reassortment events) [109]. If these novel influenza A viruses acquire the ability to transmit efficiently between humans, they may cause a pandemic outbreak [110, 111]. Seasonal influenza A and B virus infections impose a substantial annual disease burden, causing an estimated 1 billion infections, including 3–5 million severe cases, and 290,000–650,000 respiratory related deaths each year [112]. Mortality for influenza A viruses is highest in children under 5 years old and older adults over 65 years of age [47, 112]. In contrast, the highest disease burden for influenza B viruses is reported in children [113]. Influenza virus-specific CD8^+^ T cells elicited by past seasonal influenza viruses provide crucial cross-reactive immunity to novel or drifted influenza strains as they recognize epitopes derived from highly conserved internal proteins (e.g. Nucleoprotein (NP) or Matrix Protein 1 (M1)) [23]. Indeed, individuals with higher precursor NP- and M1-specific CD8^+^ T cell levels were significantly less likely to develop symptomatic, PCR-confirmed influenza during the 2009 H1N1 pandemic [19, 105]. An early and robust CD8^+^ T cell response among patients hospitalized with an avian A/H7N9 influenza infection also correlated with survival, whereas fatal cases showed minimal CD8^+^ and CD4^+^ T cell responses [21]. These findings highlight the importance of harnessing broad-reactive influenza virus-specific CD8^+^ T cells as a relevant strategy for pandemic preparedness [23, 93].

Annual influenza virus exposures likely contribute to the generation and maintenance of long-lived CD8^+^ T_RM_ populations in the lung [114–116]. Indeed, mice who have been repeatedly exposed to influenza were able to maintain influenza virus-specific CD8^+^ T_RM_ populations for >450 days, whereas CD8^+^ T_RM_ cells elicited by a single infection waned within months [117]. These influenza-specific CD8^+^ T_RM_ cells provide a rapid first line of defence during secondary infections, both in controlling local viral replication and by escalating the immune response by attracting other immune cells including inflammatory monocytes [114]. Despite their distinct tissue residency and local immune surveillance, they do share some overlapping features with circulating influenza-specific memory CD8^+^ T cell populations. Studies using human lung tissue have shown a high level of overlap between TCR expressed by influenza-specific T_RM_ and circulating memory CD8^+^ T cell populations [116, 118].

As individuals age, influenza-specific CD8^+^ T cell memory persists but becomes functionally constrained [24, 61]. We recently defined the phenotypic, transcriptomic, clonal and functional changes of CD8^+^ T cells directed at the prominent HLA-A*02 : 01 restricted M1_58_-_66_ epitopes (A2/M1_58_) across the human lifespan [23]. Our research demonstrated that the reduced functionality of influenza virus-specific CD8^+^ T cells in older adults is not attributable to terminal differentiation or exhaustion but instead stems from a shift within the TCR repertoire. While the A2/M1_58_-specific TCR repertoire in children and adults is dominated by highly functional public TCRs, they gradually disappear with increasing age and are replaced by CD8^+^ T cells expressing less functional low avidity private TCRs in older adults (Fig. 3c) [22, 25]. These suboptimal private TCR signatures were directly associated with poorer proliferation, reduced polyfunctionality and decreased capability to recognize M1_58-66_ peptide variants [25]. This gradual change from public to private TCRs in the A2/M1_58_-specific TCR repertoire has also been reported by others using bulk TCR sequencing technologies [24, 98]. It is also important to note that this model is based on a single influenza epitope-specific CD8^+^ T cell response, and it remains unclear if similar age-associated shifts occur across other epitope or virus-specific CD8^+^ T cell populations.

In contrast to influenza, RSV infections often induce relatively weak and short-lived antibody responses which eventually result in reinfection [119, 120]. Furthermore, RSV also does not induce durable, high-quality CD8^+^ T cell memory, and protective CD8^+^ T cell responses wane rapidly after infection [92, 121]. These relatively short-lived immune responses contribute to lifelong susceptibility to reinfection and symptomatic disease. The majority of individuals experience RSV infection at least once by the age of two, and ~10% of adults are reinfected each year [121, 122]. However, RSV causes substantial disease burden in infants and older adults, who experience severe lower-respiratory disease and higher rates of hospitalization [121]. Worldwide an estimated 33.1 million children under the age of 5 suffer from RSV-associated acute lower respiratory infections each year, including 3.6 million RSV-associated hospitalizations and >100.000 deaths, particularly among those <6 months old [123, 124].

CD8^+^ T cells have been shown to contribute to RSV clearance and in some cases may be associated with lung immunopathology [92, 125]. In an RSV human challenge study, Jozwick *et al*. investigated the systemic and local RSV-specific CD8^+^ T cell responses in adults. They observed extensive lower airway inflammation with persistent viral antigen and cellular infiltrates, despite rapid clinical recovery. They reported high RSV-specific T_RM_ population in convalescence without continued proliferation. Interestingly these RSV-specific CD8^+^ T_RM_ cells are less polyfunctional compared to circulating RSV-specific CD8^+^ T cells or influenza-specific CD8^+^ T_RM_ cells. RSV-specific CD8^+^ T_RM_ cells express high levels of either IFN-γ or IL-2 depending on their specificity, while polyfunctional influenza-specific CD8^+^ T_RM_ cells often also express TNF [92]. Mouse models have demonstrated that exacerbated disease was unique to RSV and not observed in influenza or SARS-CoV-2. The excessive CD8^+^ T cell response was not epitope dependent and occurred despite a significant reduction in viral titres and was likely caused by the rapid production of IFN-γ by CD8^+^ T cells [126]. Higher IFN-γ-producing circulating T cells were also found in patients with severe RSV disease requiring hospitalization [126]. Together these studies show that influenza, SARS-CoV-2 and RSV-specific CD8^+^ T_RM_ cells are functionally different.

While RSV neutralizing antibody titres are comparable between healthy young and older adults, lower numbers of functional RSV-specific CD8^+^ T memory cells are observed in older individuals [126, 127]. While more research is needed, this may suggest that susceptibility for severe RSV in older individuals is not caused by immunopathology due to a too strong or unregulated immune response, as observed in younger individuals. Instead, severe RSV in older individuals may result from waning of RSV-specific CD8^+^ T immunity and instead results in an inadequate immune response [126, 127]. This is something that should be kept in mind when designing a T cell-based RSV vaccines.

Together, influenza and RSV illustrate that both the virus itself as well as human ageing reshapes the extent of virus-specific CD8^+^ T cell immunity to viruses causing repetitive acute infections in fundamentally different ways. In influenza, ageing erodes the TCR repertoire quality, resulting in the loss of highly functional CD8^+^ T cells. In RSV, ageing exacerbates already weak and short-lived CD8^+^ T cell immunity, contributing to poorer viral control and greater susceptibility to severe lower-respiratory disease. These contrasting patterns highlight that ageing does not impair all acute viral CD8^+^ T cell responses uniformly and underscores the need for virus-specific COP and tailored intervention strategies, particularly for older adults.

### Immune responses to persistent latent viral infections

While RSV and influenza illustrate the impact of ageing on CD8^+^ T cell responses to acute and recurrent viral infections, they represent only part of the immune pressures experienced across the human lifespan. Viruses causing chronic or persistent latent infections such as CMV, EBV and VZV impose continuous or episodic antigenic stimulation that progressively reshapes CD8^+^ T cell differentiation, TCR repertoire diversity and functionality (Fig. 3d) [128]. These lifelong herpesvirus infections amplify many hallmarks of immunosenescence and create a CD8^+^ T cell landscape that is fundamentally altered over time [128].

CMV establishes lifelong latency with intermittent reactivation, and has a profound effect on T cell immunity [129]. Repeated antigenic stimulation during latency drives memory inflation, whereby CMV-specific CD8^+^ T cells expand persistently and can occupy up to 10% of the total circulating CD8^+^ T memory compartment in the blood [8, 130, 131]. Even in the context of other persistent latent virus infections, CMV seems to occupy an unusually large fraction of the memory population, often even orders of magnitude greater [8]. Phenotypically, these inflated CMV-specific memory CD8^+^ T cell populations consist predominantly of terminally differentiated T_EMRA_ cells that frequently express markers associated with senescence, including CD57 and KLRG1, while also expressing inhibitory receptors such as PD-1 [130]. The functional properties of CMV-specific CD8^+^ T cells are also affected, including reduced proliferation and polyfunctionality despite retaining cytotoxic signatures [26].

Additionally, CMV seropositivity is associated with contraction of naïve and T_CM_ populations, which results in further narrowing of the TCR repertoire [94, 132]. Furthermore, CMV has been shown to contribute to chronic low-grade inflammation associated with ageing, termed inflammaging [133, 134]. Latent CMV infection further amplifies this inflammatory baseline, as recurrent low-level reactivation and NFκB activation sustains cytokine production (IL-6, TNF, IL-1β and IL-10) and tissue stress [129]. Finally, CMV results in a conserved expansion of GZMK^+^ CD8^+^ T cells, which were identified as a hallmark of inflammaging [134, 135]. Together these studies underscore how CMV accelerates or even exceeds the effects of chronological ageing alone [129, 132] and may further limit the CD8^+^ T cell capacity to respond to both previously encountered acute and chronic or persistent latent viruses as well as novel or mutated viruses [136, 137].

For instance, CMV seropositivity is associated with a less diverse EBV-specific CD8^+^ T cell TCR repertoire, reducing the number of unique TCRs and shifting epitope dominance beyond what is attributable to ageing alone [138]. Indeed, older CMV^+^ adults show markedly blunted EBV-specific CD8^+^ T cell responses, in contrast to CMV⁻ older adults whose EBV-specific CD8^+^ T cell frequencies actually increase with age, demonstrating a selective suppressive effect of CMV on coresident EBV-specific CD8^+^ T cell immunity [139]. In addition, van den Berg *et al*. observed that CMV seropositive older individuals exhibit substantially lower baseline frequencies of influenza virus-specific memory CD8^+^ T cells compared to seronegative older individuals [140]. This may be a direct result of the CMV-driven memory inflation occupying a disproportionate share of the memory compartment but could also suggest that CMV compresses the long-lived memory population specific for (repetitive) acute viruses [140]. However, despite reduced baseline memory in older CMV seropositive individuals, their influenza virus-specific CD8^+^ T cell frequencies, function, phenotype or TCR repertoire diversity were not negatively impacted during an acute influenza infection [140].

Souquette and Thomas have proposed that lifelong exposures to viruses that cause latent or smouldering chronic infections remodel the CD8^+^ T cell landscape through mechanisms such as bystander activation, altered activation thresholds, cross-reactive TCR selection and shifts in immunodominance hierarchies [141]. According to this model, latent herpesviruses such as CMV continuously influence the cytokine environment, stromal signalling and antigen-presentation networks, thereby shaping how new CD8^+^ T cell responses are primed and maintained. Their model helps explain why persistent latent CMV infections have a disproportionate influence on responses to other acute and chronic/latent viral infections [141].

Other lifelong herpesviruses, including EBV and VZV, likewise affect the ageing CD8^+^ T cell compartment, albeit through a more subtle and mechanistically distinct pathway. Unlike CMV, which establishes latency across many cell types, EBV latency is restricted to B cells, resulting in less chronic antigen stimulation [26, 38]. Consequently, EBV-specific CD8^+^ T cell responses expand only modestly, maintain a more diverse TCR repertoire than CMV-specific populations, and can persist for decades [38]. In adults, EBV-specific CD8^+^ T cell populations are typically dominated by T_EM_ and T_EMRA_-like phenotypes [26, 38]. With increasing age, EBV-specific CD8^+^ T cells progressively adopt a terminally differentiated T_EMRA_ phenotype (including CD57^+^ and KLRG1^+^), and display reduced cytokine production and proliferative capacity [138]. Although limited heterologous cross-reactivity between EBV and Human Simplex Virus (HSV)-1 and/or HSV-2 has been reported, these cross-reactive cells exhibit suboptimal functional capacity. This indicates that cross-reactivity does not necessarily translate into effective protection or memory inflation [142].

Like EBV, VZV persists in latency for decades. Primary infection typically occurs during childhood, resulting in Varicella or chickenpox. Following the acute phase, VZV persists in sensory dorsal root and cranial nerve ganglia. A total of ~10–20% of individuals with a latent VZV infection experience reactivation later in life, often after the age of 55, leading to a painful condition known as shingles [49]. Control of latency relies heavily on VZV-specific CD8^+^ T cells. Reduced frequencies and functional impairment of VZV-specific effector-memory CD8^+^ T cells in combination with waning tissue-resident surveillance in ganglia have been associated with reactivation [49]. Why reactivation occurs in only a subset of VZV positive individuals is multifactorial. Age-related erosion of VZV-specific CD8^+^ T_EM_ and T_RM_ populations, comorbidities (such as diabetes and kidney disease), immunosuppression and host genetic factors (including certain HLA types) collectively lower the threshold for reactivation in susceptible individuals [143, 144].

These studies show that there is a complex interplay between the ageing immune system and persistent latent herpesviruses. While ageing immunity may result in losing control of a persistent latent VZV infection, there is limited evidence that VZV itself directly impacts the ageing immune response. In contrast, EBV drives ageing of the EBV-specific CD8^+^ T cell response, but by itself has limited impact on immunosenescence as a whole. This can change when EBV is part of a heterologous co-infection with CMV. CMV has the largest impact on the immune system, increasing immunosenescence, which does not only affect the CMV-specific CD8^+^ T cell population but also CD8^+^ T cells directed against other viruses.

### CD8^+^ T cell responses to novel viruses

The introduction of a novel virus into an immunological naïve population, for example in case of a pandemic outbreak, poses a unique set of challenges to the immune system.

In contrast to previous influenza pandemics, where older individuals benefited from some pre-existing humoral and/or cellular immunity [19, 91, 104, 105, 145–149], the COVID-19 pandemic provided a unique opportunity to study the generation of *de novo* T- and B cell priming in immunological naïve populations across the human lifespan. Pre-2019 sera showed robust neutralizing IgG to seasonal coronaviruses (OC43/HKU1), but failed to neutralize SARS-CoV-2, indicating low antigenic overlap [150]. Systems-serology profiling of pre-pandemic serological samples from healthy individuals across the human lifespan demonstrated moderate levels of cross-reactive, non-neutralizing antibodies against SARS-COV-2. While children exhibited adaptable IgM-rich and Fc-functional antibody repertoires, older adults displayed class-switched yet rigid, species-specific antibody networks shaped by decades of endemic coronavirus exposure [151]. Low sequence homology between SARS-CoV-2 and circulating endemic coronaviruses likely limited the extent of pre-existing cross-reactive CD8^+^ T cell memory among the human population [85, 152, 153]. However, cross-reactive T cell responses were reported among some unexposed individuals [154]. While most individuals lacked pre-existing SARS-CoV-2-specific immunity at the start of the COVID-19 pandemic, there were profound age-related differences in disease outcomes [155]. In addition, robust, broad and polyfunctional SARS-CoV-2-specific CD8^+^ T cell responses were associated with reduced disease severity and enhanced viral clearance, while severe infections were associated with qualitatively impaired CD8^+^ T cell responses [20, 78, 156–160]. This underscores a possible association between age-related differences in cellular immunity and disease outcome.

Household studies demonstrated that children were able to control a SARS-CoV-2 infection rapidly and locally [161], often resulting in less severe and often asymptomatic infections [155]. SARS-CoV-2 exposed children who were asymptomatic or displayed only mild symptoms and repeatedly tested PCR negative mounted robust salivary anti-S1 IgA, systemic Fc-functional IgG and displayed early CD8^+^ T cell activation, demonstrating near-sterilizing mucosal immunity [161]. In a larger household cohort, infected children showed profound depletion of circulating monocytes and dendritic cells, likely reflecting rapid innate recruitment to airway tissues [162, 163]. Combined with the Fc-functional antibody features observed in children, these findings indicate that early innate mobilization and a more flexible and mucosal antibody response contributed to milder paediatric disease [151, 161, 162]. In addition, several studies confirmed that seroconverted children were capable of generating T cell memory following infections, albeit with distinct features compared to adults [163–165]. While the magnitude of the spike-specific CD8^+^ T cell response in seroconverted children was comparable to adults, markedly lower CD8^+^ T cell responses to internal epitopes were observed in seroconverted children [163–165]. On an epitope level, the SARS-CoV-2-specific CD8^+^ T cells in children and adults shared common TCR features. However, the SARS-CoV-2-specific TCR repertoire in children remained more diverse and comprised fewer clonal expansions [164]. Phenotypically, the SARS-CoV-2-specific CD8^+^ T cell population in seroconverted children was skewed towards the less differentiated and long-lived T_SCM_ phenotype, whereas adults displayed a greater proportion of T_CM_ cells [156]. These findings are in line with observations for influenza virus-specific CD8^+^ T cell populations in children [25]. However, in contrast to influenza, *ex vivo* analysis and *in vitro* stimulations demonstrated that the level of SARS-CoV-2-specific CD8^+^ T cell activation was reduced in children compared to adults [25, 163, 165]. Together these findings demonstrated that the rapid viral clearance in children had a profound impact on their CD8^+^ T cell population. The reduced magnitude and functionality of their SARS-CoV-2-specific CD8^+^ T cells may seem less ideal. Yet, the less differentiated phenotype and more diverse TCR repertoire also suggest that children may have generated a longer-lasting immune response which may maintain better capacity to recognize potential epitope escape variants.

In contrast, it has frequently been suggested that the induction of a *de novo* CD8^+^ T cell responses in older individuals may be hindered by reductions in naïve CD8^+^ T cell frequencies, lower TCR repertoire diversity, impaired priming signals and accumulation of inflated or immunosenescent memory phenotypes. This would leave older individuals particularly vulnerable during a pandemic outbreak [26]. Indeed, SARS-CoV-2-specific naïve CD8^+^ T cells from health unexposed older individuals did display a lower magnitude and functionality following 10 days of *in vitro* priming compared to healthy adults [166]. *In vitro* priming from naïve T cells is a technically challenging process limited by the relatively low SARS-CoV-2-specific precursor populations at the start of the assay. Additionally, the peptide pools were not matched with the HLA of the donor and under-represented for immunodominant ORF1ab epitopes [166]. In contrast, van den Dijssel *et al*. quantified SARS-CoV-2-specific CD8^+^ T cells directly *ex vivo* in a convalescence cohort using heterotetramer combinatorial coding across a broad epitope panel in HLA-matched donors [137]. They demonstrated that the magnitude of the SARS-CoV-2-specific immune response for all 35 epitopes was maintained across age (22–66 years), was unaffected by the individual’s CMV status and could be maintained for over 200 days after infection [137]. Furthermore, a prominent T_CM_ phenotype was observed in SARS-CoV-2-specific CD8^+^ T cells in adults and older individuals [137]. These findings were confirmed by others who studied a smaller set of epitopes [167]. Together, these studies demonstrated that older and CMV positive individuals could generate a robust and broad CD8^+^ T cell memory directed against an emerging virus despite an overall smaller naïve T cell population. Furthermore, these studies seem to indicate that CMV-specific memory inflation did not compress the formation of SARS-CoV-2 CD8^+^ T cell memory in older CMV positive individuals. Further research is needed to investigate whether SARS-CoV-2-specific CD8^+^ T cells in older and/or CMV positive individuals express similar TCRs. Although Peng *et al*. did not take into account the age of their donors, they did reveal that severe SARS-CoV-2 was associated with lower avidity TCR repertoires [168].

The COVID-19 pandemic revealed that there are indeed age-associated differences in immunity to a novel virus in an immunologically naïve population. Strong early innate responses, flexible IgM-rich antibody repertoires and robust mucosal control likely protected children from severe disease. This rapid viral clearance of a first viral encounter early in life may have contributed to the formation of a broader and possibly longer-lived SARS-CoV-2-specific CD8^+^ T cell response [151, 161, 162]. Conversely, while older individuals were capable of mounting a broad CD8^+^ T cell response, an imperfect coordination across innate, humoral and cellular compartments may ultimately have resulted in severe disease outcomes [18]. These insights argue for age-tailored pandemic vaccine strategies.

### Limitations to harnessing CD8^+^ T cells through vaccination in older individuals

Effective CD8^+^ T cell responses are central to protection against severe disease outcomes, particularly from respiratory viruses that undergo rapid antigenic evolution. Yet most vaccines are designed to elicit humoral immunity. The mRNA (BNT162b2 and mRNA-1273) and vector-based (ChAdOx1-S) COVID-19 vaccines were designed to induce protective humoral immunity to the spike protein but also elicited robust T cell immunity [169–173]. Yet, several studies reported reduced vaccine effectiveness and reduced CD8^+^ T cell responses in older populations [173–176].

In a cohort study spanning ages 22–99 years, Palacios-Pedrero *et al*. demonstrated that two doses of the BNT162b2 mRNA vaccine elicited spike-specific antibodies, T cell responses and B cell memory all decreased with increasing age. The highest frequency of individuals who failed to mount a detectable T cell response (non-responders) was observed among adults≥66 years [177]. Poor responsiveness in this group correlated with reduced frequencies of naïve cells among the CD4^+^ and CD8^+^ T cells, along with increased frequencies of CD57^+^KLRG1^+^ senescent CD8^+^ T cells, which are all hallmarks of immunosenescence [177]. Notably, these age-related deficits were most pronounced for responses to novel antigens, whereas recall responses to influenza or infection-induced SARS-CoV-2 immunity were relatively preserved [177]. Further studies by Bredholt *et al*. demonstrated that an additional third BNT162b2 booster vaccination 10 months after the initial dose elicited robust neutralizing and spike-specific antibody responses in older individuals similar to those observed in younger cohorts [175]. Yet, no increase in cytokine-producing T cells was observed in older individuals following third BNT162b2 booster vaccine, which was associated with their exhausted phenotype [175].

A complementary platform comparison by Dallan *et al*. revealed that different vaccine platforms can be combined to overcome some of the age-associated challenges to induce robust immunity in older adults. Primary vaccination with BNT162b2 (mRNA) generated stronger spike-specific antibodies but weaker spike-specific CD8^+^ T cell responses, whereas ChAdOx1-S priming elicited more robust CD8^+^ T cell immunity but modest antibody titres [178]. Subsequent mRNA boosting enhanced humoral and cellular immunity in both groups, with the greatest benefit observed in older adults who initially received the ChAdOx1-S vaccine [178]. The authors reported that primary BNT162b2 vaccination was associated with erosion of the naïve T cell population, which may have limited the magnitude of the booster-induced spike-specific responses [178]. This study highlights how different vaccine platforms can be combined to overcome some of the age-associated challenges to induce robust immunity in older adults. Additional studies are needed to determine whether combining different vaccine platforms also enhances the longevity of functional SARS-CoV-2-specific T cells in older adults.

Current COVID-19 mRNA and adenoviral vaccines only target the spike protein. However, the SARS-CoV-2 spike protein is known to elicit relatively subdominant CD8^+^ T cell responses compared to epitopes derived from internal viral proteins in convalescent individuals [172, 179]. This relatively low immunodominance may have contributed to the less robust T cell immunity observed in older individuals. Incorporating non-spike SARS-CoV-2 proteins into the COVID-19 mRNA vaccine may improve long-term protection against severe disease and drifted variants [179]. Indeed, non-spike protein mRNA vaccines induced robust and broader cellular immunity capable of protecting animals from severe disease, even in the absence of neutralizing antibodies [180]. Whether these more immunodominant epitopes can indeed elicit more robust cellular immune response in older individuals remains to be established.

These novel vaccine platforms are also of interest for generating robust and lasting protection against other viruses, such as influenza. Influenza virus-specific CD8^+^ T cells recognize conserved internal proteins that are far less susceptible to antigenic drift and shift than the surface glycoproteins targeted by neutralizing antibodies [109, 181, 182]. This makes CD8^+^ T cell immunity a crucial component for influenza pandemic preparedness.

Unfortunately, most current influenza vaccines are designed to boost humoral rather than CD8^+^ T cell immunity. Furthermore, by effectively preventing infection with seasonal influenza viruses at an early age, current vaccines may also hamper heterosubtypic CD8^+^ T cell immunity otherwise elicited during natural infection [183]. This further highlights the need for alternative vaccine platforms capable of inducing robust CD8^+^ T cell immunity [93]. The question is whether mRNA vaccines would be as effective for influenza as they were for SARS-CoV-2. While there are multiple mRNA-based influenza vaccines in development, their target antigen is the HA protein [184]. It is important to note that the influenza HA protein is known for its inability to elicit robust CD8^+^ T cell responses, as it does not contain any known conserved CD8^+^ T cell epitopes [181]. The holy grail would be a universal influenza vaccine, capable of eliciting a broad protective immune response against diverse influenza virus strains and subtypes. Hereto vaccines could combine an antigenically optimized HA protein [185] with an internal influenza protein which harbours multiple immunodominant and conserved T cell epitopes (e.g. M1 and/or NP) [93]. However, in contrast to SARS-CoV-2, most humans already have pre-existing immunity towards influenza, including memory CD8^+^ T cells. It is therefore very likely that influenza mRNA vaccines will behave differently across age groups compared to COVID-19 vaccines. Timing of novel T cell-based influenza vaccines could be crucial for inducing optimal immunity. While T cell-based influenza vaccines may effectively boost CD8^+^ T cell populations expressing highly functional public TCRs in children and adults, it remains to be tested whether they will be as effective in older individuals who have a predominant less-functional private TCR repertoire [25]. While providing T cell-based influenza vaccines earlier in life may be more effective, vaccine-induced influenza-specific CD8^+^ T cell immunity will need to be closely monitored over time, as repeated vaccination may inadvertently accelerate TCR repertoire ageing [22, 25]. However, it is important to note that this hypothetical concern should be weighed against the well-established benefits of childhood influenza vaccination in preventing severe disease and reducing influenza-associated morbidity and mortality.

Taken together, these studies highlight the benefits but also some remaining challenges for T cell-based vaccine platforms to reach their full potential in older individuals [25, 177, 178]. Heterologous prime-boost strategies, particularly adenoviral-vector priming followed by mRNA boosting, may balance robust T cell immunity with high-quality humoral responses, particularly in older individuals [178]. Even so, T cell-based vaccines may ultimately perform best when administered earlier in life, when public clonotypes are abundant and the naïve TCR repertoire is most flexible to recognize a broad range of different epitopes. Conversely, late-life vaccination will need to prioritize restoring or stabilizing memory quality, through platforms that preferentially expand CD8^+^ T_CM_ and T_SCM_ cells expressing high-quality TCRs, rather than highly differentiated T_EMRA_ cells.

### Innovative strategies to improve CD8^+^ T cell-based vaccine platforms

The SARS-CoV-2 pandemic has demonstrated that both vector-based and mRNA vaccine platforms elicit robust humoral and cellular immunity. However, mRNA vaccines have two notable advantages. Firstly, unlike vector-based vaccine platforms, mRNA-based vaccines are not compromised by pre-existing or vaccine-induced anti-vector immunity. This eliminated the concerns about pre-existing vector-specific immunity interfering with vaccine effectiveness. It also means that mRNA vaccines can be used to elicit immunity to a range of different viruses across the human lifespan. Secondly, mRNA vaccines can be designed in a way that they encode additional immunomodulatory molecules which may further boost the virus-specific immune response without the need of external adjuvants [72, 186–189]. Despite these two major advantages, it remains likely that mRNA vaccines will need to be tailored to different situations to generate the most robust CD8^+^ T cell response. This might be dependent on both the virus and the age group.

To elicit *de novo* CD8^+^ T cell immunity, derived from naïve CD8^+^ T cells in case of a pandemic outbreak or in young children, mRNA vaccines may be combined with type I IFNs (associated with effective priming of naïve CD8^+^ T cells) or IL-7, IL-15 and IL-21 cytokines (associated with pro-survival properties of CD8^+^ T cells) [72, 186]. In contrast, the addition of IL-12 has been shown to improve the expansion of memory T cells and could be used to boost existing acute virus-specific memory CD8^+^ T cell populations [186]. Conversely, exhausted chronic or latent virus-specific CD8^+^ T cells may be rejuvenated by combining the vaccine with checkpoint inhibitors such as anti-PD1 [189, 190]. Alternatively, exhausted CD8^+^ T cells may also benefit from ablating their RASA2 expression, which has been shown to improve functionality of exhausted cancer cells [191]. While these approaches show promise for enhancing CD8^+^ T cell immunity, many remain experimental and are currently supported primarily by preclinical studies or observations from cancer immunotherapy. Further work is required to determine their safety and efficacy in the context of vaccination against viral infections.

Taken together, these studies demonstrate that there are multiple ways to improve CD8^+^ T cell immunity through vaccination, which may be further tailored to benefit the ageing population.

### Conclusions, future directions and challenges

Lifelong viral exposures shape CD8^+^ T cell immunity in age- and virus-specific ways. Acute, recurrent, chronic or latent and novel infections, as well as age-associated changes such as thymic involution, loss of naïve T cells, TCR repertoire narrowing and altered memory-subset composition, all progressively constrain the breadth, flexibility and functional coordination of virus-specific CD8^+^ T cell responses. These constraints have direct implications for the protection against severe disease outcomes in older adults, particularly in the absence of neutralizing antibodies.

The field would greatly benefit from systematic efforts to define COP for CD8^+^ T cells. Well-defined COP could help us better identify high-risk populations and more accurately monitor vaccine-induced CD8^+^ T cell immunity over time. Even though we have a relatively good idea of the CD8^+^ T cell properties that could serve as a COP, standardized assays to measure these responses and clearly defined protective thresholds remain elusive. There are several reasons for this: (1) CD8^+^ T cells control disease outcomes rather than infection, which makes COP harder to define; (2) Both precursor frequency and the functional quality of the different phenotypic subsets serve important roles in predicting clinical outcome. A simple measurement of frequency does not capture the functional and phenotypic heterogeneity that contributes to the level of protection; (3) Tissue-resident populations play an important role in protecting against severe disease, especially for respiratory viruses, but are difficult to access and monitor; (4) The high level of HLA diversity within the human population means that different individuals recognize different viral epitopes, making it particularly hard to generate an assay that captures a universal protective threshold. This is further complicated by the fact that immunodominance of epitopes changes in the context of different HLA settings or following repeated exposures [137, 172], (5) What further complicates the identification of uniform COP for CD8^+^ T cells is the presence of age-associated changes within the CD8^+^ T cell population; and finally, (6) COP are shaped by both virus-specific aspects and the presence of chronic or persistent latent viral co-infections.

Moving forward, longitudinal datasets are needed to track how potentially CD8^+^ T cell COP, including magnitude, phenotypic composition, polyfunctionality, epitope breadth and TCR diversity evolve after vaccination using different vaccine platforms, dosing intervals and delivery strategies (e.g. mucosal versus systemic) [25, 177, 178]. Such frameworks will be essential for designing and fine-tuning vaccines that remain effective within the constraints imposed by immunosenescence and by the lifelong viral exposures that shape the ageing immune system.

## Abbreviations

A2/M1_58_: HLA-A*02:01 restricted M1_58-66_ epitope
CMV: cytomegalovirus
COP: correlates of protection
COVID-19: Corona virus disease 2019
EBV: Epstein–Barr virus
GZMK: Granzyme K
HA: Haemagglutinin
HLA: Human Leukocyte Antigen
HPV: human papillomavirus
HSV: Herpes Simplex Virus
IFN-γ: Interferon gamma
IL: Interleukin
M1: Matrix protein 1
NA: Neuraminidase
NKκB: Nuclear factor-κB
NP: Nucleoprotein
ORF1ab: Open reading frame 1ab
PCR: polymerase chain reaction
pHLA-I: peptide bound to a HLA class I molecule
RSV: respiratory syncytial virus
SARS-CoV-2: severe acute respiratory syndrome coronavirus 2
T_CM_: Central Memory T cell
TCR: T cell receptor
T_EFF_: Effector T cell
T_EM_: Effector memory T cell
T_EMRA_: Terminally differentiated effector memory T cells
T_naïve_: Naïve T cell
TNF: Tumor necorsis factor
T_RM_: Resident memory T cell
T_SCM_: Stem-cell memory T cell
VZV: varicella-zoster virus
